# Organizational health culture in the Korean firefighter intervention studies: a scoping review

**DOI:** 10.3389/fpubh.2025.1537976

**Published:** 2025-04-15

**Authors:** Hanbit Jin, Hyungsun Jun, Jisu Ha, Inae Youn, Jungtae Leem

**Affiliations:** ^1^Department of Diagnostics, College of Korean Medicine, Wonkwang University, Iksan, Republic of Korea; ^2^Department of Preventive Medicine, College of Korean Medicine, Dongshin University, Naju, Republic of Korea; ^3^Department of Acupuncture and Moxibustion, National Medical Center, Seoul, Republic of Korea; ^4^Research Center of Traditional Korean Medicine, College of Korean Medicine, Wonkwang University, Iksan, Republic of Korea; ^5^Department of Il-won Integrated Medicine, Wonkwang University Korean Medicine Hospital, Iksan, Republic of Korea

**Keywords:** Republic of Korea, firefighter, intervention study, organizational health culture, scoping review

## Abstract

**Introduction:**

This study examines the current state of intervention studies focused on Korean firefighters, who face unique health challenges due to the demands of their work and specific organizational culture. Recognizing a gap in studies tailored to firefighters’ needs, this scoping review aimed to identify existing interventions and provide recommendations for future research.

**Methods:**

Following the Joanna Briggs Institute’s methodological guidelines for scoping reviews and the PRISMA-ScR checklist, a seven-stage review was conducted. The search included articles from Korean and international journals published up until January 25, 2024. Data were analyzed using a pre-developed framework and the Template for Intervention Description and Replication checklist. This study was registered on the Open Science Framework (OSF) and is accessible at https://osf.io/s378j/.

**Results:**

From 35 articles analyzed, results showed an imbalance in topics, research design, outcome measures, and intervention delivery methods. Additionally, most studies lacked specific focus on firefighters’ unique organizational characteristics and needs.

**Discussion:**

The study highlighted three areas for enhancing intervention research quality: designing and tailoring studies to fit the specific demands of firefighting, ensuring ethical considerations, and aligning with the practical needs of firefighters. Future research should prioritize diverse interventions addressing firefighters’ health, incorporate sensitive and ethical research designs, and work closely with experts in firefighting to develop adaptable and relevant interventions.

**Systematic Review Registration:**

https://osf.io/s378j/, Registration DOI: https://doi.org/10.17605/OSF.IO/7EQ8M.

## 1 Introduction

Firefighters are employed in a complex occupational culture, characterized by a number of specific job characteristics and organizational climates. The job characteristics of firefighters can be summarized as follows: “hazardousness,” “uncertainty,” “shift work,” “urgency,” and “role ambiguity” (1). The term “hazardousness” refers to job characteristics involving various risk factors such as physical injuries and psychological trauma that occur during job performance. Uncertainty implies that disasters are inherently uncertain (2), necessitating firefighting organizations to be on standby 24/7 to respond to unforeseeable disasters. Further, Article 13 of the “Firefighter Duty Regulations” (Work Method of 119 Safety Centers) (3) states that shift work should typically follow a three-shift system (three shifts, two rotations). However, in cases where firefighting conditions render this impractical, a two-shift system (two shifts, one rotation) may be used if the workload permits. The attribute of urgency implies that in urgent situations such as fires and disasters, swift dispatch and arrival at the scene are crucial for minimizing disaster spread (4), necessitating firefighters to maintain alertness even during standby. Unpredictable situations occur repeatedly during emergency dispatches and on-site activities; however, the individual discretion of firefighters is limited. Role ambiguity arises when there is inadequate clarity regarding roles or information about a scene (5). This has a significant negative impact on job performance, particularly in complex job roles such as firefighting (6).

The job characteristics of firefighters are not universally applicable. Firefighting organizations perform various tasks, including responding to disaster scenes, emergency treatment and transportation of patients, disaster prevention, computational work, communication, and emergency reporting tasks. Therefore, individualized psychological and social interventions targeting firefighters are essential because the personal situations of individuals and their environments differ (7). However, individualized interventions carry the risk of disclosing personal information, necessitating a careful research design. Identifying at-risk groups and providing interventions can involve stigmatization; therefore, caution is necessary. Firefighters are often perceived as emphasizing trustworthiness and robustness because of the nature of their profession. This perception leads to concerns about experiencing disadvantages or being perceived as weak because of mental health treatment (8). Moreover, firefighting organizations are characterized by hierarchical organizational climates that prioritize rank in the performance of hazardous duties. If elements of prejudice and discrimination against the vulnerable are tolerated in such organizational climates, stigmatization may occur (9). The social condemnation and decreased self-esteem associated with stigma have been demonstrated to have deleterious effects on individuals’ reputations and professional careers (10). Consequently, interventions targeting firefighters should be designed considering the job characteristics and organizational climate of firefighting organizations.

Firefighters are frequently exposed to environments that can cause physical injuries and psychological trauma. The uncertainty of firefighting duties has been shown to negatively affect job performance, sleep patterns, and social/familial relationships (11); moreover, shift work-related circadian rhythm sleep–wake disorders can significantly impair mental and physical health (12). Furthermore, the urgency of firefighting tasks to meet the golden hour for firefighting, rescue, and emergency medical activities can lead to circadian rhythm disruption and stress (13). A systematic review on non-cancerous health risks among firefighters (14) found that firefighters have a higher risk of mental disorders, hearing impairment, myocardial infarction, and ischemic stroke than the general population. The International Agency for Research on Cancer has classified firefighting as a Group 1 carcinogen owing to the elevated risk of cancer associated with occupational exposure among firefighters. Additionally, firefighters frequently sustain injuries during urgent on-site activities, with musculoskeletal injuries such as tension, sprains, and muscle pain accounting for 36% of the cases (15). These health issues among firefighters have been demonstrated to negatively impact their job performance (16). Consequently, firefighter health issues extend beyond individual concerns to encompass socio-economic losses (17), underscoring the importance of implementing interventions to address these health concerns.

These issues necessitate structured programs tailored to the characteristics of firefighters for prevention, treatment, and case management. Intervention studies are necessary to develop and evaluate these programs. While numerous intervention studies targeting Korean firefighters have been conducted, most have been group-based face-to-face programs. However, they have been criticized for not adequately reflecting the characteristics of firefighters and the demands of their work environment (18). Group-based face-to-face programs are typically conducted in the workplace during working hours. In such cases, there is often a lack of safe and comfortable spaces outside the workplace for conducting counseling sessions with high-risk groups for specific conditions, which may result in participants refusing to participate (19). Additionally, practitioners of the “Visiting Counseling Center for Firefighters,” organized by the National Fire Agency, have often found it challenging to meet research participants due to urgent deployments (20). Another issue, as evidenced by a study targeting 119 emergency medical technicians, is that while most fire stations (88.3%) operate health management programs, they focus on operating fitness facilities or holding special lectures, thus lacking diversity (21). Problems with health management programs include programs that do not meet the demands of the participants, one-time operations, and coercion to participate, resulting in low satisfaction (21). From the perspective of firefighter health and safety policies, ensuring anonymity among users of mental-health-related programs has emerged as a significant issue (22). Although numerous literature reviews have been conducted on the health problems of Korean firefighters, most have focused on specific areas such as mental health and psychological intervention studies (19, 20), post-traumatic stress disorder (PTSD) (23–25), and suicidal ideation (26); thus, limiting their scope. Moreover, no literature review has examined whether these studies reflect firefighter characteristics.

The objectives of this study were as follows: first, to ascertain the current status and characteristics of intervention studies for firefighters in Korea; second, to determine the extent to which intervention studies for firefighters in Korea reflect the organizational culture of firefighters; and third, to propose recommendations for designing intervention studies for firefighters in Korea. Furthermore, we aim to conduct a series of research projects in the future to identify the unmet health needs of firefighters and promote their health through a combination of qualitative research (27), survey research, and retrospective chart reviews.

## 2 Methods

This study employed a scoping review methodology (28) to understand the current status and characteristics of all intervention studies, including mental and physical interventions, targeting Korean firefighters. Furthermore, the study examined the extent to which the study design took into account the organizational culture of firefighters. The scoping review methodology comprises seven steps, based on the methodological guidance provided by the Joanna Briggs Institute (29). Furthermore, we followed the Preferred Reporting Items for Systematic Reviews and Meta-Analyses Extension for Scoping Reviews (PRISMA-ScR) checklist (30) for reporting. The protocol for this study was registered on the Open Science Framework and can be accessed at https://osf.io/s378j/.

### 2.1 Review questions

The following research questions will be addressed in this study:

1. What is the current status of intervention studies on Korean firefighters published in academic journals since 2010?
2. With respect to the aforementioned studies, to what extent have their study designs considered firefighters’ organizational culture?

### 2.2 Inclusion criteria

The inclusion criteria were as follows: (1) Literature published in academic journals after 2010; (2) Articles reporting interventions; (3) Studies in which participants include Korean firefighters and rookie firefighters; (4) No restrictions on outcome variables, study design, or language. The following document types were excluded: gray literature such as dissertations, government documents, policy reports, and conference proceedings.

### 2.3 Search strategy

A literature search was conducted from January 25, 2024 to February 1, 2024, targeting articles published in Korean and international academic journals. The databases used for the search included PubMed, Embase, Cochrane Central Register of Controlled Trials (CENTRAL), DataBase Periodical Information Academic (DBpia), Research Information Sharing Service (RISS), Oriental Medicine Advanced Searching Integrated System (OASIS), Korea Citation Index (KCI), Korean studies Information Service System (KISS), ScienceON, Korean Medical Database (KMBASE), and International Clinical Trials Registry Platform (ICTRP). Furthermore, we conducted a thorough examination of the citations and references in the existing literature. The search terms used for Korean academic journals were “firefighter,” “fire service,” and “fireman,” while “firefighter” and “Korea” were used for international journals. Based on the specified search terms, search strategies were devised according to the characteristics of each database. The detailed search strategies can be found in Supplementary Table 1.

The process of including and excluding literature was conducted independently by two researchers (HJ and HJ). In the event of a discrepancy, a consensus was reached through discussions between the researchers. If a consensus could not be reached, a third researcher (JL) was consulted for the final selection. The titles and abstracts of the studies were initially reviewed based on the inclusion and exclusion criteria. Subsequently, the full texts of the initially selected literature were examined, and a final selection was made. The reasons for excluding studies that did not meet the inclusion criteria were recorded.

### 2.4 Evidence screening and selection and data extraction

The analysis framework used in this study was developed based on the criteria used in previous literature reviews (31). The framework included publication year, author, journal information, study design, intervention type, participant age, sample size, and outcome variables. Furthermore, we extracted additional information according to the Template for Intervention Description and Replication (TIDieR) checklist (32) to improve intervention reporting.

The data extraction process was identical to the literature selection process, as conducted by all three researchers (HJ, HJ and JL). The rationale for including items in the final analytical framework and the assumptions made during data extraction are presented in Supplementary 1.

### 2.5 Data analysis and presentation of results

**Figure 1:**
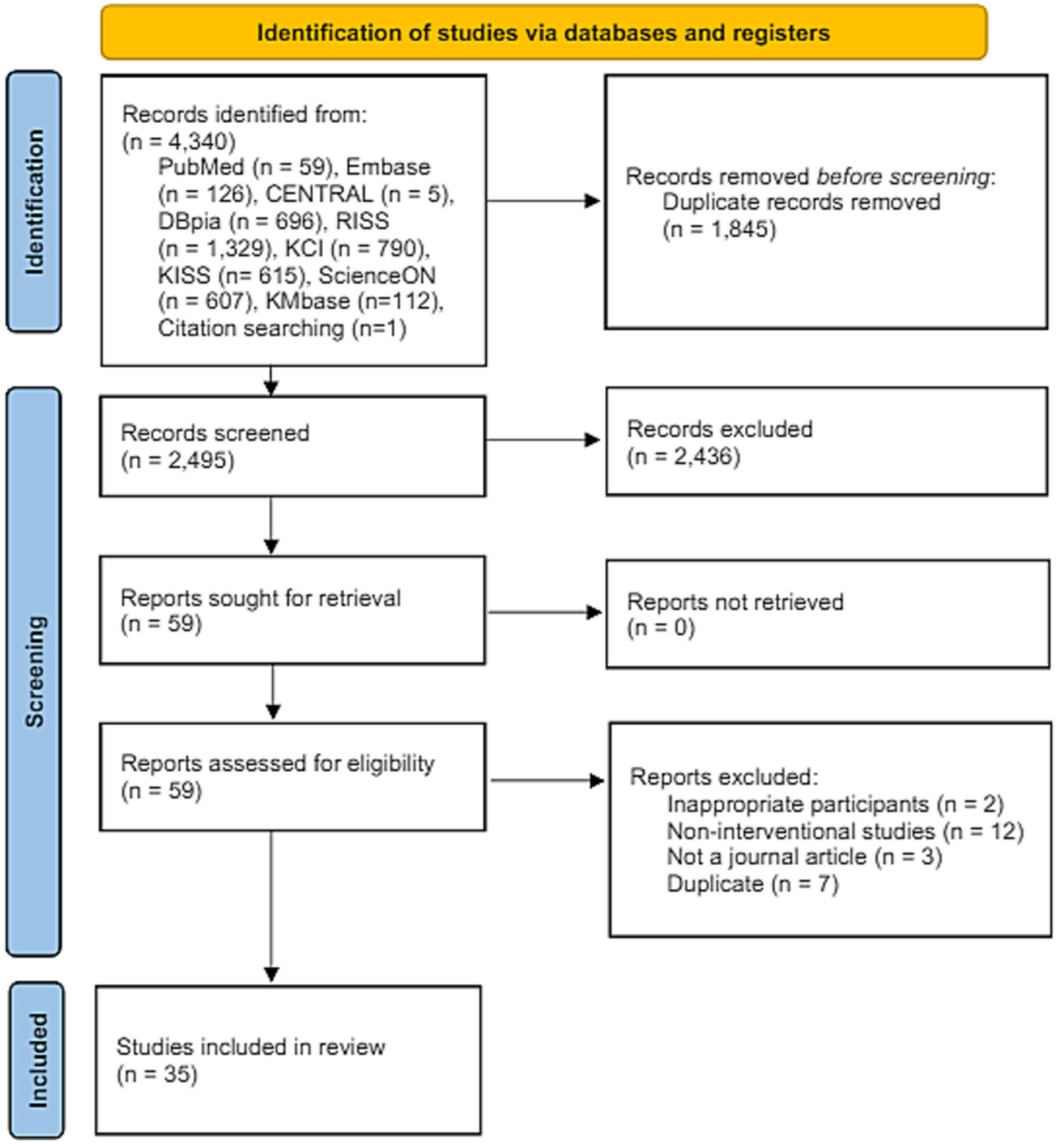
PRISMA flow diagram.

The extracted data were presented in tables or figures in accordance with the objectives of this study. Although the general characteristics of the included studies, protection of participant anonymity and confidentiality and research ethics, and data regarding the TIDieR checklist were not analyzed, they were presented in the form of tables. Subsequently, the data were supplemented through frequency and percentage analyses, descriptive summaries, and critical discussions of the research results. The literature search results were reported in the form of a flow diagram following the PRISMA-ScR guidelines (Figure 1).

## 3 Results

### 3.1 Study selection

A total of 4,340 studies were identified through database and citation searches, and the resultant data were managed using EndNote X9 software (Clarivate Analytics, Philadelphia, PA, USA). Following the exclusion of 1,845 duplicate documents, the remaining 2,495 studies underwent a first-round selection based on the inclusion and exclusion criteria, and their titles and abstracts were reviewed. Consequently, 2,436 studies were excluded, leaving 59 studies for full-text screening. Subsequently, 24 studies were excluded based on the inclusion/exclusion criteria after reviewing the full text, resulting in a final sample of 35 studies (Figure 1).

### 3.2 General characteristics of the included studies

Table 1 presents the general characteristics of the 35 studies in the final sample. The publication years were divided into three categories: 2010–2014 (*n* = 3; 8.5%), 2015–2019 (*n* = 16; 45.7%), and 2020–2024 (*n* = 16; 45.7%). Given that only one publication from 2024 was included in the search, the trend appears to have gradually increased over time. The number of participants in the included studies ranged from 12 to 1,859. Regarding research design, quantitative studies were the most prevalent at 29 studies (82.9%), including 2 randomized controlled trials (5.7%), 10 quasi-experimental studies (28.6%), and 17 pre-experimental studies (48.6%). Further, while five studies (14.3%) followed a mixed-methods design (14.3%), only one (2.9%) had a qualitative design. The majority of the selected studies (*n* = 27; 77.1%) employed psychological and social interventions. With respect to outcome measures, 9 studies (25.7%) used objective measurements, while 26 (74.3%) used self-report questionnaires.

**Table 1:** General characteristics of included studies.

Year_Author	Intervention	Outcome	Sample size	Sex	Age		Firefighter classification	Duration of service	
True-experimental study									
2019_Baek (35)	Korean red ginseng†	PSS, POMS, SDS, CPT, Subjective cognitive impairment (65), Vital Sign, Blood test	E: 32➔28 C: 31➔27 Normal C: 37	E(M:17, F:15) C(M:15, F:16)	E: 39.6 C: 41.3		Nurses and firefighters	NR	
2024_Kim (47)	The virtual mate exercise training program	Body composition, Aerobic capacity, Isokinetic muscular function	E:10➔9 C:10	M:20	E: 36.4 ± 5.9 C: 40.1 ± 8.0		NR	NR	
Quasi-experimental study									
2013_Nam (63)	Stress management program	Way of Coping Checklist, Job stress (4), Symptom Checklist-90-Revised	E: 26➔21 C: 26➔20	E(M:21, F:0) C(M:17, F:3)	E ≤ 39 s: 10 40s: 10 ≥ 50s: 1	C ≤ 39 s: 10 40s: 7 ≥ 50s: 3	Rescue First aid Administration	E ≤ 3: 2 3–10: 9 > 10: 10	C ≤ 3: 4 3–10: 5 > 10: 11
2014_Park (66)	Emotional Freedom Technique Program	Stress Response Inventory, IES-R	E: 41 C: 44	E(M:41) C(M:44)	E: 42.2 C: 41.6		Firefighting Rescue, First aid Administration	E: 14.2 C: 14.4	
2015_Shim (41)	Music therapy†	Post-trauma Risk Checklist	E: 21 C: 21	E(M:12, F:9) C(M:10, F:11)	E 20–35: 6 35–39: 8 over40: 7	C 20–35: 8 35–39: 8 over40: 5	Firefighting: 18 Rescue & First aid: 24	E < 10: 5 10–15: 8 ≥ 15: 8	C < 10: 5 10–15: 10 ≥ 15: 6
2016_Baek (67)	Music listening†	Korean Occupational Stress Scale -SF	88➔80 E: 40 C: 40	E(M:36, F:4) C(M:35, F:5)	E 20s: 12 30s: 17 40s: 5 50s: 6	C 20s: 12 30s: 14 40s: 9 50s: 5	Firefighting: 31 Rescue: 8 First aid: 21 Administration: 20	E ≤ 3: 8 3–10: 21 ≥ 10: 11	C ≤ 3: 7 3–10: 20 ≥ 10: 13
2016_Lee (68)	Protective equipment†	Physical, Biochemical, Perceived	12	M:12	35.1 ± 1.5		NR	7.1 ± 6.4	
2017_Paik (62)	Group arts psychotherapy	Korean Occupational Stress Scale, Way of Coping Checklist, Person in the Rain	E: 10 C: 10	E(M:10) C(M:10)	E 20s: 1 30s: 2 40s: 3 50s: 4	C 20s: 1 30s: 2 40s: 3 50s: 4	Firefighting: 6 First aid: 10 Administration: 2 Team leader: 2	E ≤ 5: 2 6–10: 2 10–20: 1 ≥ 20: 5	C ≤ 5: 3 6–10: 0 10–20: 3 ≥ 20: 4
2018_Han (7)	PTSD Management Program†	PCL-5, PHQ-9	E: 25➔22 C: 25➔22	E(M:20, F:2) C(M:21, F:1)	E ≤ 30s: 0 30s: 8 40s: 8 ≥ 50s: 6	C ≤ 30s: 3 30s: 5 40s: 10 ≥ 50s: 4	Firefighting: 17 Rescue: 5 First aid: 16 Administration: 6	E ≤ 5: 2 6–10: 4 11–14: 3 ≥ 15: 13	C ≤ 5: 2 6–10: 5 11–14: 2 ≥ 15: 13
2020A_Lee (34)	Integrative art therapy program	Meaning in Life Questionnaire	E: 54 C: 54	E(M:42, F:12) C(M:41, F:13)	NR		Prospective firefighters	0	
2022_Hur (46)	Educational program	Interpersonal Reaction Index, Self-Reflection Scale (69), RCS	E: 20 C: 20	E(M:11, F:9) C(M:16, F:4)	20s: 4, 30s: 3 40s: 5, 50s: 4		Firefighting: 18 Rescue: 5 First aid: 17	NR	
2023A_Kim (70)	Two new Cardiopulmonary Resuscitation (CPR) training methods	CPR performance knowledge, performance practice, self-efficacy, Class Immersion, Class Satisfaction	Medical VR: 64➔60 Flipped learning: 64➔61	Medi-VR (M:58, F:6) Flipped (M:58, F:6)	Medi-VR: 28.4 ± 3.1 Flipped: 27.8 ± 3.8		Firefighting: 109 First aid: 12	NR	
Pre-experimental study									
2014_Chung (45)	Eye Movement Desensitization and Reprocessing†	Clinician-Administered Post-traumatic stress disorder Scale	15➔7	NR	NR		NR	NR	
2016_Kim (71)	Critical Incident Stress Debriefing	Way of Coping Checklist, IES-R	30	M:29, F:1	30s: 10 40s: 9 50s: 11		NR	<5: 1 5–10: 8 10–15: 1 ≥ 20: 20	
2017_Kim (37)	Mental health program	PCL-S, BDI, BAI, SSI, ISI	679➔502	M:484, F:18	40.1 ± 8.1		NR	NR	
2017_Mo (38)	Integrated management program for PTSD	PCL-5, PHQ-9	170	M:165, F:5	20s: 5 30s: 82 40s: 45 ≥ 50s: 38		NR	≤5: 21 6–10: 75 11–20: 29 ≥ 21: 45	
2017_Lee (72)	Trauma group education	Questionnaire*	21	M:21	30s: 8 40s: 6 ≥ 50s: 7		NR	≤ 10: 10 10–20: 4 ≥ 20: 7	
2018_Lee (8)	Meditation program	PTGI, Connor-Davidson Resilience Scale	26	M:20, F:6	34.4 ± 7.4		First aid	Firefighter: 6.08 ± 6.74, First aid: 4.28 ± 4.48	
2018_Shin (39)	Camp program	PSS, Global Assessment of Recent Stress Scale, IES-R, BDI-II	90➔ F/U 1 month: 20➔ F/U 3 months: 61➔ F/U 6 months: 39	M:82, F:8	41.1 ± 9.1		Firefighting: 35 Rescue: 10, First aid: 26 Administration etc.: 19	<10: 34 10–20: 27 > 20: 29	
2019_Park (73)	Forest therapy program	PCL-5, POMS-B	293➔196	M:186, F:10	20s: 14, 30s: 48 40s: 59, 50s: 67 60s: 8		NR	<10: 62 10–20: 43 20–30: 73 ≥ 30: 18	
2020B_Lee (42)	Personalized bird sounds	Vital signs for heart, kidney, lungs	24	M:20, F:4	20s: 12 30s: 9 40s: 3		Firefighting: 8 Rescue: 5, First aid: 6 Command and investigation: 5	NR	
2020_Kim (74)	High-Intensity Interval Training (HIIT) program	Body composition, Cardiovascular endurance, Muscle endurance, Muscle power, Job performance	16	NR	35.76 ± 8.75		NR	NR	
2020_Jang (43)	Sleep intervention	Sleep diary, ISI, DDNSI, ESS, DSI-SS, PHQ-9, PCL-5	45➔39	M:33, F:6	43.33 ± 9.32		NR	9.98 ± 7.62	
2020_Won (19)	Mental health promotion program	PCL, BDI, BAI, BSS, ISI, AUDIT, WHOQOL-BREF	1,859	M:1,757, F:97	40.75		NR	NR	
2021_Jang (75)	Experience of the care farm	Salivary cortisol, Perceived Restorativeness Scale, ES-R, PSS, Program satisfaction	16	M:14, F:2	36.3 ± 7.96		Firefighting: 12 Rescue: 1 First aid: 3	<1: 2 1–5: 10 5–10: 1 ≥ 10: 3	
2022_Park (64)	Hoegi-type forest healing program	Korean Occupational Stress Scale, IES-R, Center for Epidemiological Studies-Depression Scale	72➔63	NR	20s: 9 30s: 25 40s: 20 50s: 9		Firefighting: 23 Rescue: 6 First aid: 32 Administration: 2	<5: 17 5–10: 15 10–15: 13 15–20: 5 ≥ 20: 13	
2022_Cha (36)	Forest-based trauma stress management program†	Job stress (76), California Personality Inventory, ES-R, Symptom Checklist-90-Revised, Salivary cortisol	23➔20	M:17, F:3	39.3 ± 10.0		Firefighting: 10 Rescue: 1 First aid: 6 Administration: 3	(Months) 114 ± 123.1	
2022_Lee (77)	Four interventions for promoting voluntary exercise training†	Mean minutes of exercise training per week, Three salient beliefs (78), Intervention reaches dose fidelity	175➔99	NR	NR		Firefighting Rescue First aid	NR	
2023B_Kim (44)	Meditation-based healing program†	ISI	23➔20	M:18, F:2	42 ± 11.1		Firefighting: 5 Rescue: 1, First aid: 5 Administration: 7 Dispatcher: 2	12.69 ± 10.30	
Mixed method study									
2018_Yook (40)	Body-psychological exercise program	WHOQOL, IES-R, Physical Symptom Scale (79), Somatosensory Amplification Scale, Individual interview materials	E: 12➔11 C: 12➔11	NR	E: 43.7 ± 12.6 C: 44.3 ± 10.4		Firefighting: 5 Administration: 1 Firefighting, Rescue, First aid: 10 Protection: 1 No response 5	NR	
2019B_Kim (33)	Meditation–based healing program	IES-R, Event-Related Ruminating Inventory, Program review	80	M:40, F:40	M: 41.9 ± 11.2 F: 36.9 ± 09.0		Firefighting: 30 Rescue: 10 First aid: 40	≤ 10: 49 10–20: 10 ≥ 20: 20	
2019A_Kim (80)	Nomex body cooling garment	Completion time, Heart rate, Total sweat rate, Blood lactate acid, Individual interview materials	16	M:16	36.7 ± 8.0		NR	9.5 ± 7.5	
2023_Bak (81)	Simsang-poetry therapy program	PSS, SDS, KRQ-53, Oral and technical records	E:15➔9 C: 9	E(M:8, F:1) C(M:8, F:1)	E: 44 C: 47		NR	E: 16 C: 16	
2023_Kwak (18)	Online post-traumatic growth program†	Event-Related Ruminating Inventory, SSS, PTGI, Focus Group Interview materials	E: 17➔16 C: 17	NR	E: 33.6 ± 4.0 C: 33.9 ± 5.2		Firefighting: 2 First aid: 30 Administration: 1	(Months) E: 72.4 ± 41.4 C: 79.1 ± 47.8	
Qualitative study									
2022_Yoon (82)	Happy art therapy	Individual interview materials, Program review	787	M:623, F:164	20s – 50s		NR	NR	

* No references found, †Provide specific inclusion criteria.

AUDIT, Alcohol Use Disorders Identification Test; BAI, Beck Anxiety Inventory; BDI, Beck Depression Inventory; BSS, Beck Scale for Suicidal ideation; C, Control group; CPT, Continuous Performance Test; DDNSI, Disturbing Dream and Nightmare Severity Index; DSI-SS, Depressive Symptom Inventory-Suicidality Subscale; E, Experimental group; ESS, Epworth Sleepiness Scale; F, Female; F/U, Follow Up; IES-R, Impact of Event Scale-Revised; ISI, Insomnia Severity Index; KRQ-53, Korean resilience quotient-53; M, Male; NR, Not Reported; PCL-5, Post-traumatic Stress Disorder Checklist for DSM-5; PHQ-9, Patient Health Questionnaire-9; POMS-B, Profile of Mood States-Brief; PSS, Perceived Stress Scale; PTGI, Post-traumatic Growth Inventory; RCS, Relationship Change Scale; SDS, Sheehan Disability Scale; SF, Short Form; SSI, Scale for Suicidal Ideation; SSS, Social Support Scale; WHOQOL, World Health Organization Quality of Life.

In order determine whether firefighters received tailored interventions, the characteristics of the participants were examined in terms of sex, age, firefighter classification, and length of service. Ten studies (28.6%) provided specific inclusion/exclusion criteria. Among the included studies, six that did not report sex were excluded. An examination of the remaining studies revealed that the participants comprised 4,108 males (89.5%) and 484 females (10.5%), with no studies designed to include individuals of other genders. Six studies exclusively involved male firefighters, while none involved female firefighters exclusively. However, one study implemented different interventions based on gender during certain stages of the program (33). The ages of the study participants ranged from 20s to 60s, and no study specifically targeted a particular age group. Studies targeting specific firefighter classifications included one focusing on first aid personnel who participated in a mindfulness meditation program (8), one evaluating an online trauma growth program for firefighters with more than 3 years of first aid service (18), and one implementing integrated art therapy for rookie firefighters (34). Additionally, one study included both nurses and firefighters from high-stress occupations to investigate the effects of red ginseng (35). The length of service of firefighters ranged from less than 1 year to over 20 years. Studies that included length of service as a criterion for participant selection included one study targeting first aid personnel with over 3 years of service (18), one study targeting individuals with over 3 years of first aid service (36), and one study implementing integrated art therapy for rookie firefighters (34).

### 3.3 Protection of participant anonymity and confidentiality and research ethics

Information regarding participant protection and research ethics for the final selection of 35 studies are presented in in Supplementary Table 2. Among the selected studies, 15 (42.9%) reported obtaining Institutional Review Board (IRB) approval, and 26 (74.3%) mentioned obtaining participant consent. Seven studies (20%) identified high-risk groups, with five (71.4%) mentioning IRB approval and all seven (100%) mentioning obtaining participant consent. Additionally, six studies (85.7%) mentioned details regarding financial support, indicating a higher frequency with regard to ensuring participant anonymity and confidentiality and research ethics compared to studies that did not classify high-risk groups. In the final sample of 35 studies, 15 (42.9%) explicitly stated that they had received financial support, while only one (2.9%) explicitly stated that they had not received financial support. Nineteen studies (54.3%) did not specify whether the authors had received financial support.

### 3.4 TIDieR checklist

The description and replication of the interventions included in the final sample of 35 studies were extracted according to the TIDieR checklist, and the results are presented in Supplementary Table 3. Among the 34 quantitative and mixed-methods studies, 16 (47.1%) divided the participants into experimental and control groups when implementing the interventions. Further, six qualitative and mixed-methods studies were identified, employing various methodologies, including phenomenological analysis, content analysis, case study, live experiential study, focus group interviews; one study had an unspecified methodology. Among the 35 included studies, four involved researcher-led interventions and 15 involved interventions by other professionals, such as therapists.

To ascertain how the research design of the final 35 studies reflected the characteristics of firefighters’ work, data were extracted using the TIDieR checklist. The results are summarized in Supplementary Table 4. Fifteen studies (42.9%) involved interventions within fire stations, whereas 13 (37.1%) involved interventions outside fire stations. One study did not specify the intervention location, and six studies did not describe it. A total of 13 studies (37.1%) conducted interventions during working hours, whereas 13 (37.1%) conducted interventions outside working hours. One study did not specify the timing of the intervention, while seven studies did not describe it. Tailored interventions were implemented in 12 studies (34.3%). These included six studies that employed individual counseling (7, 19, 37–40), two studies that used musical instruments or bird sounds based on participants’ preferences (41, 42), two studies that provided individual sleep goals and personal management strategies (43, 44), one study that adjusted the frequency of intervention based on participants’ improvement status (45), and one study that conducted different interventions for men and women during certain program stages (33).

The most prevalent mode of intervention delivery was the face-to-face group intervention, employed in 19 studies (54.3%). Thirty studies (85.7%) exclusively used face-to-face interventions, three studies (8.6%) exclusively provided non-face-to-face interventions, and two studies (5.7%) employed a combination of face-to-face and non-face-to-face methods. Among the non-face-to-face intervention studies, one employed online lectures (46), another conducted online lectures and anonymous group counseling via social networking services (18), and another used a virtual mate exercise training program instead of an exercise instructor (47). Group-only interventions were the most common, comprising 21 studies (60%), followed by individual-only interventions in 6 studies (17.1%). Seven studies (20%) employed a combination of group and individual interventions, whereas one (2.9%) did not specify the intervention format.

## 4 Discussion

### 4.1 Background of the study

A common feature of firefighting organizations in Korea and other countries is that most countries have a hierarchical class system and a paramilitary culture, and are developing into comprehensive emergency services that include rescue and first aid in addition to firefighting. However, in terms of a state-led integrated firefighting organization, South Korea is more centralized than the United States and the United Kingdom, and, in contrast to the United States and Germany, is focused on professional firefighters. In contrast, the United States and Germany have a high proportion of volunteers. On the other hand, it has similarities with Japan in terms of a central-local dual structure and a comprehensive scope of work, but the difference is that the scale of volunteer fire brigades in Japan is much larger. Therefore, Korean firefighters are greatly influenced by the policies of the central government. In Korea, firefighters generally work approximately 84 h per week, and 14.3% of firefighters have been exposed to occupational diseases. However, due to evaluations related to the payment of bonuses, 88% of firefighters who have suffered injuries have not declared them as official diseases (48).

In Korea, there has been a growing awareness of the importance of firefighter welfare, as evidenced by the enactment in 2012 of the Framework Act on Health, Safety and Welfare of Firefighting Officials, the Human Rights Status Survey of Korean Firefighters in 2015 (48), the conversion of local firefighters to national civil servants in 2020, and the establishment of a national fire hospital (scheduled for completion by 2025). However, there is a paucity of studies on Korean firefighters that have been published in international journals. Furthermore, in contrast to the extant literature reviews on Korean firefighters, which has been published exclusively in Korean journals on the topic of mental health, research on firefighters is actively being conducted abroad and various literature reviews have been published. Literature reviews on diseases, such as the systematic literature review on non-cancer health risks of firefighters (14) and the systematic literature review on cancer incidence in firefighters (49), research is active on a range of topics, including a literature review on the prevention and management of infections among emergency medical service workers (50), a literature review on rehabilitation (51), and a literature review on the training of firefighters and injuries during training (52). However, there has been no literature review on whether the intervention study for firefighters considered organizational health culture. Consequently, there is a necessity to systematically organize related studies and to inform the international community of the Korean case, with a view to filling the academic gap and suggesting future research directions.

### 4.2 Design and content

Although the number of intervention studies on firefighters has been increasing steadily, there has been a notable imbalance in terms of the research topics, study designs, outcome variables, and forms of intervention delivery. This review indicated that Korean firefighters are at an elevated risk of mental health issues, with numerous intervention studies being conducted to address this issue. However, the principles of mental health promotion programs outlined by the World Health Organization and International Labor Organization indicate that they should be comprehensive, in conjunction with other health promotion programs to be effective. Firefighters who have experienced PTSD exhibit significantly higher rates of cardiovascular, respiratory, musculoskeletal, and nervous system symptoms than those who have not (53). Since mental health problems often manifest as biological symptoms, such as insomnia or pain, maintaining biological health is crucial for mental health management (54). Most of the studies included in this review focus on psychological and social interventions to address mental health issues. In future research, it is essential to design intervention studies that employ a multidimensional approach to address mental health issues among firefighters in Korea and integrate them with other health promotion programs.

It is crucial to conduct high-quality intervention studies using optimal experimental designs. Randomized controlled trials (RCTs) are the gold standard for evidence-based research, in which participants are randomly assigned to experimental and control groups. This methodology ensures that the two groups are homogeneous except for the intervention, which allows for causal inference. However, RCTs require significant resources, such as human resources and costs, which are often unavailable in research settings. Therefore, it is essential to provide research funding for conducting RCTs. Moreover, increased research funding is essential to actively implement RCT methodology in future research, thereby boosting the quality of intervention studies on firefighters.

The most prevalent format of intervention delivery among the included studies was the group-based face-to-face format (*n* = 19, 54.3%), with only four studies (11.4%) conducting follow-up observations. Given that firefighters are repeatedly exposed to traumatic incidents, repeated training over multiple sessions is necessary to enhance psychological resilience and proficiency in required skills (55). It is essential to develop a range of intervention formats tailored to the specific research context and intervention type rather than relying on a single uniform group-based face-to-face approach. Furthermore, research designs that incorporate both self-report questionnaires and objective evaluation indicators are necessary. Studies involving long-term follow-up observations to assess the long-term effects of interventions and case management of research participants are also needed.

### 4.3 Tailoring and ethics

Firefighters are exposed to different risks depending on their assigned tasks (56–58), working periods (59), and gender (60), and the resulting health issues vary, making individual health issues heterogeneous within the group. Moreover, firefighting is among the occupations with the highest prevalence of mental health issues requiring case management (61). Scholars have opined that underscoring the necessity for tailored interventions over uniform ones. However, a limitation of existing studies is the lack of targeting specific firefighter groups in the selection criteria of the included studies. It is essential to provide evidence-based interventions by referencing past studies, pilot studies, or expert consensus when designing the research and selecting appropriate participants.

Furthermore, sensitive research designs that consider appropriate intervention methods, timing, and location are essential to accommodate participants’ needs. In group-based, face-to-face programs, it is of paramount importance to ensure that populations that are high-risk or discriminated against are not singled out. Among the five studies that identified high-risk groups and obtained IRB approvals, three explicitly addressed participant protection methods. If the identification of high-risk groups or discrimination experiences is intended, all stages of the program should target the entire participant group, with individual feedback on diagnostic results and research progress. In instances where this is not a viable option, alternative methodologies such as online or virtual reality-based interventions, in conjunction with anonymization techniques employing pseudonyms or other means, can safeguard the privacy of participants. Furthermore, if the study design identifies high-risk groups or those who have experienced discrimination as the sole participants, it may be necessary to consider alternative modalities of intervention delivery. For instance, the intervention could be delivered individually or virtually, rather than in a group-based face-to-face program.

### 4.4 Organizational culture and demands of the field

Despite the numerous intervention studies targeting Korean firefighters, these studies have failed to adequately reflect the irregular duty hours and demands of fieldwork, including irregular emergency response times and shift work. Among the 35 studies included in this review, various opinions regarding suitable intervention locations and timings were identified through post-intervention reflections. The limitations of studies that conducted interventions within fire departments during the workday were described as follows: ‘The intervention was not easily implemented in an environment where we had to wait for an emergency response. The sirens and emergency response affected the implementation of and participation in the intervention (62),’ ‘We were unable to see a significant difference in outcome variables because we had to leave in the middle of the intervention due to unexpected work events (63),’ and ‘It was not uncommon to have to return to the site without meeting the participants due to emergency response (20).’ Overall, interventions conducted at fire stations during working hours encountered challenges in separating firefighter duties from intervention provisions, making it difficult to assess intervention effectiveness. Therefore, future interventions should aim to increase intervention focus by separating intervention time and location from work responsibilities. The limitations of studies that conducted interventions outside of the fire station during off-duty hours were described as follows: ‘Conducting the intervention after work may have the physical and psychological burden on participants (64)’ and ‘Using personal time, such as holidays, may have reduced participation and effectiveness (7).’ Due to the nature of shift work among firefighters, providing interventions during personal time after long shifts may burden the participants and reduce intervention effectiveness. Moreover, given the varying shift schedules of firefighters, it may be challenging to gather all participants at designated times. Overall, future efforts should focus on improving working conditions such as ensuring sufficient staffing for shift work or providing interventions directly at fire stations during working hours. Future research should investigate the intervention timing and locations that maximize the effectiveness of interventions targeting firefighters.

The preceding recommendations are reflections based on researchers’ perspectives through the review of the intervention studies. There is a paucity of research analyzing the opinions of firefighters—the research participants—through qualitative studies to identify solutions. According to a study (18), there is a need for flexibility to accommodate individual and organizational characteristics of firefighters, practical content provided by firefighting experts, avoidance of outcome orientation, and inclusion of positive content rather than focusing solely on negative aspects. This study used a flexible research design that allowed participants to select the most comfortable time and space outside their working hours. Additionally, to avoid stigma, the study conducted anonymous online counseling rather than an uncomfortable psychological program. In this manner, the intervention was tailored to the needs of the participants and involved experts in firefighting psychology, demonstrating a deep understanding of firefighting.

### 4.5 Limitation

The study focused on publications in Korean and international journals since 2010; thus, there may have been studies published before 2010 that were excluded, including theses, government documents, policy reports, and conference proceedings. Furthermore, as only intervention studies targeting Korean firefighters were included, the findings may not apply to other countries and regions.

## Data Availability

The original contributions presented in the study are included in the article/Supplementary material, further inquiries can be directed to the corresponding authors.
