# Supplementary material for: Organizational health culture in the Korean firefighter intervention studies: a scoping review
Source: Front Public Health. 2025 Apr 15;13:1537976. doi: 10.3389/fpubh.2025.1537976 (PMC12037558; doi:10.3389/fpubh.2025.1537976)
Supplement: Supplementary file 1 [file Data_Sheet_1.docx]

Supplementary Material

## Supplementary

**Supplementary 1.** Rationale for including items in the final analytical framework and assumptions made in the data extraction

## Supplementary Tables

**Supplementary Table 1.** Search strategy by database

**Supplementary Table 2.** Participant protection and research ethics

**Supplementary Table 3.** Intervention description and replication of the included studies

**Supplementary Table 4.** Studies that considered firefighters’ job characteristics

**Supplementary 1. Rationale for including items in the final analytical framework and assumptions made in the data extraction**

The inclusion of items in the final analysis framework was based on the following reasons. Financial support in intervention studies has been found to correlate with research quality, and transparency regarding conflicts of interest necessitates the disclosure of funding sources (1). Furthermore, methods that identify individuals at high risk of developing a disease without participant protection are more likely to result in stigmatization than to be accurate, and thus should be avoided (2). However, there are conflicting opinions regarding the rapid identification of high-risk groups for suicide prevention among firefighters (3). Therefore, we examined whether high-risk groups were identified in intervention studies to understand how this process was conducted. It is of utmost importance that ethical considerations, including Institutional Review Board (IRB) approval, participant consent, and confidentiality, are given due consideration in intervention research. Therefore, we conducted a thorough examination of the ethical considerations of the reviewed studies. The extent of firefighters' exposure to traumatic incidents varies according to their years of service, rank, and job classifications (4). A longer service duration is associated with a higher risk of musculoskeletal disorders (5). Additionally, the proportion of female firefighters worldwide is low (6), and the research on female firefighters is limited (7). Therefore, we extracted data on firefighter classifications, years of service, and sex to assess whether the interventions were tailored to individual characteristics.

In accordance with the Template for Intervention Description and Replication (TIDieR) checklist, the additional data extracted included the following: brief name, what (material, procedure), who provided, how, where, when and how much, tailoring, and how well. These data were extracted to provide a comprehensive description regarding the intervention and its replicability. The quality of the included studies was not evaluated. To conduct effective intervention studies, it is necessary to have an environment in which the interventions can be targeted towards specific participants, without affecting their work duties (8). However, owing to the irregular duty hours and shift work characteristics of firefighters, interventions are often provided within fire stations during working hours, which can negatively affect the execution of interventions and participation (8). Conversely, scholars have also opined that providing interventions outside of working hours may result in lower participation rates and effectiveness (9). Consequently, the “Where” and “When” items of the TIDieR checklist were examined to ascertain whether the intervention was provided in a stable environment distinct from the workplace and whether it was provided during non-work hours.

The research design was classified into three categories: quantitative, qualitative, and mixed-method studies. Quantitative studies were further divided into three subtypes: true experimental, quasi-experimental, and pre-experimental.

The research fields of academic journals were classified according to the Web of Science categories for international journals, with preference given to classifications with higher Journal Impact Factor percentiles when multiple classifications were present. For Korean journals, the classification was based on the KCI classification system.

The categorization of firefighters' positions included the following: firefighting, rescue, first aid, and administrative. Data that did not fit these categories were described in terms of the original data of the paper.

When average age and length of service were provided with standard deviation, these values were extracted. For studies where these values were not provided, the original data were extracted.

The classification of high-risk groups was based on whether specific participants were classified into high-risk groups and received different interventions.

Ethical considerations were categorized based on whether Institutional Review Board (IRB) approval was obtained and whether consent was obtained from participants.

For questionnaire-based studies, only the original questionnaire was extracted. For the "Where" item of the TIDieR checklist, interventions provided outside the fire station were considered as provided outside working hours. The timing of outcome measurements was categorized as follows: pre-intervention, during intervention, post-intervention, and follow-up observations.

**Supplementary Table 1.** Search strategy by database

| Database | Search Details | Results |
| --- | --- | --- |
| PubMed | ("Firefighter"[Text Word] OR "Fire and Rescue Personnel"[Text Word] OR "Fire Fighters"[Text Word] OR "Fire Fighter"[Text Word] OR "Fire Fighters"[Text Word] OR "fire man"[Text Word] OR "Firefighter"[Text Word] OR "firefighters"[Text Word] OR "firefighting"[Text Word] OR "fireman"[Text Word] OR "Fire Fighter"[Text Word] OR "firefighters"[MeSH Terms]) AND ("korean"[Text Word] OR "korean ethnic group"[Text Word] OR "Koreans"[Text Word] OR "korean people"[Text Word] OR "korea"[Text Word] OR "South Korea"[Text Word] OR "korea republic of"[Text Word] OR "korea republic of"[Text Word] OR "korea south"[Text Word] OR "Republic of Korea"[Text Word] OR "Seoul"[Text Word] OR "South Korea"[Text Word] OR "Republic of Korea"[MeSH Terms]) AND 2020/01/01:3000/12/31[Date – Publication] | 59 |
| Embase | ('fire and rescue personnel':ab,ti,kw OR 'fire fighters':ab,ti,kw OR 'fire man':ab,ti,kw OR 'firefighter':ab,ti,kw OR 'firefighters':ab,ti,kw OR 'firefighting':ab,ti,kw OR 'fireman':ab,ti,kw OR 'fire fighter':ab,ti,kw OR 'fire fighter'/exp) AND ('korean':ab,ti,kw OR 'korean (ethnic group)':ab,ti,kw OR 'koreans':ab,ti,kw OR 'korean (people)':ab,ti,kw OR 'korean (people)'/exp OR 'korea':ab,ti,kw OR 'south korea':ab,ti,kw OR 'korea, republic of':ab,ti,kw OR 'korea, south':ab,ti,kw OR 'republic of korea':ab,ti,kw OR 'seoul':ab,ti,kw OR 'south korea':ab,ti,kw OR 'south korea'/exp) AND [2000-2024]/py | 126 |
| CENTRAL | #1 'Firefighter':ab,ti,kw OR 'Fire and Rescue Personnel':ab,ti,kw OR 'Fire Fighters':ab,ti,kw OR 'Fire Fighter':ab,ti,kw OR 'fire fighters':ab,ti,kw OR 'fire man':ab,ti,kw OR 'firefighter':ab,ti,kw OR 'firefighters':ab,ti,kw OR 'firefighting':ab,ti,kw OR 'fireman':ab,ti,kw OR 'fire fighter':ab,ti,kw 400  #2 MeSH descriptor: [Firefighters] explode all trees MeSH  87  #3 'korean':ab,ti,kw OR 'Korean (ethnic group)':ab,ti,kw OR 'Koreans':ab,ti,kw OR 'Korean (people)':ab,ti,kw Limits  9771  #4 'korea':ab,ti,kw OR 'South Korea':ab,ti,kw OR 'Korea, Republic of':ab,ti,kw OR 'korea, republic of':ab,ti,kw OR 'korea, south':ab,ti,kw OR 'Republic of Korea':ab,ti,kw OR 'Seoul':ab,ti,kw OR 'South Korea':ab,ti,kw Limits  10144  #5 MeSH descriptor: [Republic of Korea] explode all trees MeSH  1813  #6 #1 OR #2 Limits  400  #7 #3 OR #4 OR #5 Limits  15318  #8 #6 AND #7 with Cochrane Library publication date from Jan 2000 to Jan 2024, in Trials 5 | 5 |
| KISS | 소방공무원" or 전체 = "소방직"  or 전체 = "소방관"   발행연도 2000년~2024년 | 615 |
| DBpia | 전체=소방공무원 OR 전체=소방직 OR 전체=소방관 | 696 |
| RISS | ( 논문명 : 소방공무원 <OR> 논문명 : 소방직 <OR> 논문명 : 소방관 ) ( 전체 : 건강 | 1,329 |
| KCI | '소방공무원', '소방직', '소방관' | 790 |
| ScienceON | “전체=소방공무원 OR 전체=소방 공무원 OR 전체=소방관 OR 전체=소방직 ” | 607 |
| OASIS | '소방공무원', '소방직', '소방관' | 0 |
| KMBASE | 소방공무원" or 전체 = "소방직"  or 전체 = "소방관" | 112 |

CENTRAL, Cochrane Central Register of Controlled Trials; DBpia, DataBase Periodical Information Academic; RISS, Research Information Sharing Service; OASIS, Oriental Medicine Advanced Searching Integrated System; KCI, Korea Citation Index; KISS, Korean studies Information Service System; KMBASE, Korean Medical Database

**Supplementary Table 2.** Participant protection and research ethics

| Year_Author | Classifying high-risk groups | Purpose and protection measures | Ethical consideration | | Funding |
| --- | --- | --- | --- | --- | --- |
|  |  |  | IRB | Consent |  |
| **True-experimental study** | | | | | |
| 2019_Baek(10) | O | Study inclusion criteria is high-risk groups | O | O | O |
| 2024_Kim(12) | X |  | O | O | O |
| **Quasi-experimental study** | | | | | |
| 2013_Nam(13) | X |  | O | O | NR |
| 2014_Park(15) | X |  | X | X | NR |
| 2015_Shim(16) | O | Study inclusion criteria is high-risk groups | X | O | NR |
| 2016_Baek(17) | X |  | X | O | O |
| 2016_Lee(18) | X |  | X | X | O |
| 2017_Paik(8) | X |  | X | X | NR |
| 2018_Han(9) | O | Study inclusion criteria is high-risk groups  To ensure confidentiality, all stages of the program were conducted with all firefighters in the fire departments of the experimental group. Personal counselling was conducted on a one-to-one basis in a confidential space. | O | O | O |
| 2020A_Lee(19) | X |  | X | O | NR |
| 2022_Hur(20) | X |  | X | X | NR |
| 2023A_Kim(22) | X |  | O | O | X |
| **Pre-experimental study** | | | | | |
| 2014_Chung(23) | O | Identify and implement other interventions for high-risk groups The treatment progress and diagnosis results were communicated to individuals individually. | O | O | O |
| 2016_Kim(24) | X |  | X | X | NR |
| 2017_Kim(25) | X |  | O | O | NR |
| 2017_Mo(26) | O | Identify and follow up with high-risk groups | X | O | O |
| 2017_Lee(27) | X |  | X | X | NR |
| 2018_Lee(28) | X |  | X | O | NR |
| 2018_Shin(29) | X |  | O | O | O |
| 2019_Park(30) | X |  | X | O | NR |
| 2020B_Lee(31) | X |  | X | X | NR |
| 2020_Kim(32) | X |  | X | O | NR |
| 2020_Jang(33) | X |  | O | O | O |
| 2020_Won(34) | O | Identify and counsel high-risk groups. | O | O | O |
| 2021_Jang(35) | X |  | X | O | O |
| 2022_Park(36) | X |  | X | X | NR |
| 2022_Cha(37) | X |  | O | O | NR |
| 2022_Lee(39) | X |  | O | O | O |
| 2023B_Kim(41) | X |  | X | O | NR |
| **Mixed-method design study** | | | | | |
| 2018_Yook(42) | X |  | O | O | O |
| 2019B_Kim(44) | X |  | X | O | NR |
| 2019A_Kim(45) | X |  | O | O | O |
| 2023_Bak(46) | X |  | X | O | NR |
| 2023_Kwak(47) | O | Identify and counsel high-risk groups  The intervention was conducted face-to-face, and all participants used nicknames to ensure anonymity. | O | O | O |
| **Qualitative study** | | | | | |
| 2022_Yoon(48) | X |  | X | X | NR |

IRB, Institutional Review Board; NR, Not Reported; PTSD, Post-Traumatic Stress Disorder

**Supplementary Table 3.** Intervention description and replication of the included studies

| Year_Author | Material | Procedure | Who provided | When and how much | | When to measure | | | |
| --- | --- | --- | --- | --- | --- | --- | --- | --- | --- |
|  |  |  |  | Duration | Length*Time | Pr | D | Po | F/U |
| True-experimental study | | | | | | | | | |
| 2019_Baek(10) | E: Korean red ginseng (KRG) capsule (LAX-101) (Korean red ginseng powder was manufactured by Korea Ginseng Corporation, Seoul, Korea, from roots of a 6-year-old red ginseng, Panax ginseng Meyer, harvested in the Republic of Korea.) C: Placebo  Normal C: No action | Participants deemed eligible were randomly assigned using an Excel program by the study coordinator. Both clinicians and participants were blinded to allocation throughout. Packaging, storage, and handling for LAX-101 and placebo were identical. The normal control group received neither KRG nor placebo. | NR | 6 weeks | 4 Capsule*bid | O | X | O | X |
| 2024_Kim(12) | E: Virtual mate (VM) exercise training program (My benefit Co, Korea) C: Maintain their lifestyle without performing any extra exercise | The VM exercise program comprises 14 exercises, each lasting 60 seconds followed by a 30-second recovery period, with 5-minute warm-up and cooldown periods. In weeks 1–4, exercises like sidestep, pushup, arm walking, and others are performed, tailored for beginners. Weeks 5–8 involve more challenging exercises such as full burpee, pike pushup, and jump squat. Some exercises (e.g., two-arm dumbbell row, shoulder press) increase in intensity by approximately 20%, for example, using 10 kg dumbbells in weeks 1–4 and 12 kg in weeks 5–8. | VM device | 8 weeks | 30 min*24 | O | X | O | X |
| Quasi-experimental study | | | | | | | | | |
| 2013_Nam(13) | E: Stress management program providing cognitive behavior therapy  C: NR | 1. Why am I suffering? (Self-introduction, stress recognition O, X)  2. Who am I? (MBTI self-checking)  3. Who are you?  4. Easy to cope with the problem like this! (Role play using empty chair technique and magic shop)  5. I am the one dreaming happy change (communication skill training for solving conflict situation, letter to change myself, sharing impressions with each other) | NR | 1 month | 120 min*8 | O | X | O | X |
| 2014_Park(15) | E: EFT program C: Theory-focused education training program | 1. Pre-training measures, 2. Theoretical review, 3. EET learning, 4. EFT practice, 5. Post-training measures | NR | 1 day | 120 min | O | X | O | X |
| 2015_Shim(16) | E: Short-term music therapy C: Break time | Each session was divided into early, middle, late, and closing phases, with interventions such as improvisation, rhythm-making, nanta, and couples’ physical activity. | 2 music therapy professionals | 5 days | 60 min*10 | O | X | O | 4 wks, 12 wks |
| 2016_Baek(17) | E: Music listening C: Break time Equipment: MSIP-RMMLGE-DMSM3530 & PHILIPS AZ100W/61. | The introductory phase consisted of 5 mins of breathing and stretching, followed by 20 mins of music, ending with 5 mins of sharing feelings. Intervention used 37 songs from various genres. | NR | NR | 30 min*10 | O | X | O | X |
| 2016_Lee(18) | E1. Usual clothes E2. Firefighter protective clothing E3. Firefighter protective clothing with self-contained breathing apparatus Equipment: WNT3000i, Korea | Participants drank 5 mL/kg of water an hour before exercise for hydration. They wore cardiorespiratory sensors and experimental clothing. Exercise: 20 mins at 9 METs (6 km/h, 10% incline) Temperature: wet bulb globe temperature index of 22.6±0.9℃ with a warm air fan. | Wearing garments | 3 weeks | 20 min*3 | O | O | O | X |
| 2017_Paik(8) | E: Group arts psychotherapy C: No action | A combination of art, movement, meditation, and writing, with 10 mins of greeting and sharing, 15 mins of relaxation activities, 35 mins of art therapy program activities, and 30 mins of sharing and wrap-up. | A researcher and two graduate students | 3 months | 90 min*13 | O | X | O | X |
| 2018_Han(9) | E: PTSD management program C: PTSD education program | 1. Education, 2. Individual counseling, 3. Group therapy (Meditation, EMDR, Cognitive behavior therapy) | Researchers who are experts | 8 weeks | 300–360 min*5 | O | X | O | X |
| 2020A_Lee(19) | E: Integrative art therapy using semantic therapy program  C: Integrity and safety education | 1. Pre-test, 2. Motivational lecture and introducing my nickname, 3. Values auction, 4. Problem solving, 5. Finding my happiness with picture cards, 6. Life review, 7. Meeting with my mentor and creating my list, 8. Post-test | NR | 8 weeks | 60 min*8 | O | X | O | X |
| 2022_Hur(20) | E: Educational program for peer psychological support  C: NR | 1. Introduction, 2. Counseling psychology and counseling practice, 3. Crisis counseling | NR | NR | 120 min*12 | O | X | O | X |
| 2023A_Kim(22) | E1: Medical VR simulator based on self-directed learning (VR-based CPR education system CBS 2.0; TETRA SIGNUM, Korea) E2: Flipped learning (Teaching-learning model by the NFSA) | E1: Learning theoretical background, simulating practice (medi-VR simulation program order), and individual or collaborative team activities. E2: CPR theory and AED use condensed into a 10-min video. Uploaded to the "NFSA Flipped Learning" online space via Kakao Talk group chat, along with PowerPoint materials and promotional video exclusive to participants. | NR | 1 days | NR | O | X | O | X |
| Pre-experimental study | | | | | | | | | |
| 2014_Chung(23) | EMDR | 1. Patient history and treatment planning, 2. Preparation, 3. Assessment, 4. Desensitization and reprocessing, 5. Positive cognitive implantation, 6. Body search, 7. Closure, 8. Reassessment Stages 1 and 2 are done once, while Stage 8 is done at the start of each session. Each session involves Stages 3 to 7. | 2 psychiatrists 1 clinical psychologist | NR | 90 min*4 (Maximum, Tailoring) | O | X | O | X |
| 2016_Kim(24) | Critical Incident Stress Debriefing leader program | 1. Introduction, 2. Facts, 3. Thought, 4. Reaction, 5. Symptoms, 6. Teaching, 7. Re-entry | NR | 5 days | NA | O | X | O | X |
| 2017_Kim(25) | Mental health promotion program | 1. Introduction, 2. Plenary lecture, 3. Individual session (counseling), 4. Termination | Education: Psychiatrists Individual counseling: Psychiatrists, Mental health nurses, Clinical psychologists and Art therapists | 8 weeks | Education:  50 min Individual counseling: 30 min*2 (Minimum, Tailoring) | O | X | O | X |
| 2017_Mo(26) | Integrated management program for PTSD | 1. Prevention education and identification of high-risk individuals, 2. Individual counseling and group therapy (cognitive behavioral therapy, progressive muscle relaxation, EMDR) for early detection and treatment, 3. Case management program for follow-up | EMDR: Professionally qualified clinical psychologist Other: Researcher | 8 weeks | Education:  60 min Individual counseling: 1–4 times (Tailoring) Group:  4 times | O | X | O | X |
| 2017_Lee(27) | Trauma group education | Understanding trauma, trauma treatment and prevention, dream psychology, stress management, mental health support, success stories | NR | 3 weeks | 90 min*6 | O | X | O | X |
| 2018_Lee(28) | Mind subtraction meditation program | Lectures and meditations; includes the processes of opening the mind, knowing the mind, self-reflection, and mind subtraction. | 3 meditation experts | 3 days | NA | O | X | O | X |
| 2018_Shin(29) | Camp program | Relaxation therapy, forest therapy, art therapy, outdoor physical activity, MBTI test, group counseling, and in-depth one-on-one consultation | In-depth consultation: Psychiatrist Others: NR | 4 days | NA | O | X | O | 1wk, 3wks, 6wks |
| 2019_Park(30) | Forest therapy program | Forest therapy, psychotherapy, hydrotherapy, balance therapy, aroma therapy, healing equipment experience, safety-based therapy lectures | Lecture: Psychology lecturer Others: NR | 5 days | NA | O | X | O | X |
| 2020B_Lee(31) | Personalized bird sounds Equipment: Smartphone, Praat | 10 bird sounds from YouTube. Test done in noise-shielded room or office, smartphone 30 cm away. Restarted in emergencies to maintain result integrity. | NR | 1 day | 5 min | O | X | O | X |
| 2020_Kim(32) | High-Intensity Interval Training (HIIT) program | Warm-up: 10 mins before, cool-down: 5 mins after. Each set: 4 mins, 20s exercise/10s rest, repeated 4 times. Adaptation: 2 sessions in weeks 1–2, 3 sessions in weeks 3–6, 4 sessions in weeks 7–8, increasing intensity. 6 exercises per muscle group, no equipment, targeting chest, back, lower body, shoulders, and abs. | NR | 8 weeks | 30 min*24 | O | X | O | X |
| 2020_Jang(33) | Sleep Intervention FIT-IN | The “FIT-IN” program: 3 sessions: 2 face-to-face group sessions and 1 individual phone session. Includes sleep restriction, stimulus control, and nightmare rescripting. 1st group: Sleep education, 2nd group: Nightmare, 3rd phone: Maintaining change. | Group facilitators: Main therapist and two graduate students Telephone session: Clinical psychologist | 2 weeks | Group:  90 min*2 Telephone: 20 min | O | X | O | X |
| 2020_Won(34) | Mental health promotion program | The program included individual counseling followed by group education for all. Participants could have up to four counseling sessions if volunteered or deemed high risk. | A team of 20 professionals with diverse expertise: psychiatrists, mental health nurses, clinical psychologists, counselors, etc. | NR | Education:  60 min Individual counseling: 30 min (Maximum 4, Tailoring) | O | X | O | X |
| 2021_Jang(35) | Experience of the care farm | 1. Landscape appreciation of care farm, 2. Color food art, 3. Laughter therapy, 4. Making cheong using blueberry. | Farmers and expert lecturer | 1 day | 180 min | O | X | O | X |
| 2022_Park(36) | Hoegi-type forest healing program using urban forests | Introduction: Relaxing warm flower tea and warm-up exercises like forest gymnastics, yoga breathing, and stretching. Development: Explanation of program, precautions, and activities aligned with healing goals. Conclusion: Participants share feelings, provide positive feedback, discuss assignments, prepare for next session, then depart. | 1 Forest therapy leader 1 Assistant facilitator | 4 weeks | 120 min*4 | O | X | O | X |
| 2022_Cha(37) | Forest-based trauma stress management programs | Forest healing programs and psychology-related lectures in four areas: (1) counseling, (2) self-reflection, (3) resilience, and (4) physical health promotion. | NR | 5 days | NA | O | X | O |  |
| 2022_Lee(39) | 1.VR exercise system intervention 2.Poster intervention 3.Wearable health device intervention 4.Monitor intervention | To promote belief in exercise training, firefighters received a 15-minute program via VR and posters (VR and poster interventions). They were provided wearable health devices for peer support (wearable device intervention). Monitors in different locations displayed messages highlighting exercise's importance and colleagues' engagement (monitor intervention). | Exposed in the workplace | VR:  2 months Poster:  1 month Wearable device & Monitor:  5 months | NA | O | O | O | 1yr |
| 2023B_Kim(41) | Meditation-based healing program | Mindfulness of the self, self-understanding, mind subtraction, healing meditation | 3 Meditation experts | 2days | NA | O | X | O | X |
| Mixed method study | | | | | | | | | |
| 2018_Yook(42) | E: Mindfulness based body-psychological exercise program C: No action | Qualitative methodology: phenomenology analysis  Lecture on mindfulness theory, 20 mins practice, followed by 80 mins of mindfulness yoga: hatha yoga, breathing, meditation. Individual counseling after 1 week. | Researcher who are experts | 6 weeks | Program:  120 min*12 Individual counseling: 30 min | O | X | O | X |
| 2019B_Kim(44) | Mind subtraction meditation–based healing program | Qualitative methodology: Content analysis  Knowing how mind works, self-care meditation, and mind subtraction. | 2 Meditation experts | 3 days | NA | O | X | O | X |
| 2019A_Kim(45) | E1: Cooling vest A + FPPE (4 Cooling packs, 13.6kg) E2: Cooling vest B + FPPE (6 Cooling packs, 14.1 kg) E3: Nomex cooling shirt + FPPE (Nomex Cooling Shirt, 4 Cooling packs, 13.8 kg) C: FPPE (12.9kg) | Qualitative methodology: NR  Firefighters performed 8 tasks continuously: dragging hose, ladder setup/climbing/dismantling/transporting, rescuing, carrying gear, stairs walking. No breaks, walk fast but don't run. After each set, 5-min rest. Repeat 3 times. | Wearing garments | 3 months | 22~24 min*4 | O | O | O | X |
| 2023_Bak(46) | E: Simsang-poetry therapy program “Light of Heart” C: NR | Qualitative methodology: Lived experience research  Utilizing techniques from the book “I wish you could write your heart out” (Park Jung-Hye, 2020) Each session incorporated 1–3 techniques, with art being used for 2 sessions, poetry and imagery for 7 sessions, and writing for 12 sessions. | Researcher | 12 weeks | 120 min*12 | O | X | O | X |
| 2023_Kwak(47) | E: Online Post-Traumatic Growth Program  C: General stress management program provided by the National Fire Agency | 1. Program orientation, Building intimacy 2. Self-exposure, 3. Consider the meanings of events, 4. Manage negative emotions, Colleague support, 5. Positive life insights, Implement deliberate rumination, 6. Gratitude and wisdom, 7. Beliefs and goals, 8. Positive reevaluation | 1 Psychiatric mental health nurse practitioner 1 Level-I mental health professional 1 Research assistant | 8 weeks | 120 min*8 | O | X | O | 4wks |
| Qualitative study | | | | | | | | | |
| 2022_Yoon(48) | Happy art therapy | Qualitative methodology: Case Study  1. Warm-up, 2. Thematic activity, 3. Centering, 4. Sharing. Main techniques: dance movement therapy, art therapy, photography therapy, music therapy, and literature therapy. | 1 Program supervisor 10 Program leaders with expertise 10 Volunteers 16 Fire service administrators | NR | 90 min*12 | X | O | O | X |

AED, Automated External Defibrillator; CPR, Cardiopulmonary Resuscitation; C, Control group; D, During; E, Experimental group; EFT, Emotional Freedom Technique; EMDR, Eye Movement Desensitization and Reprocessing; F/U, Follow-Up; FIT-IN, Firefighter’s Therapy for Insomnia and Nightmares; FPPE, Firefighting Personal Protective Equipment; MBTI, Myers-Briggs Type Indicator; MET, metabolic equivalent of task; NFSA, National Fire Service Academy; NA, Not Applicable; NR, Not Reported; Po, Post; Pr, Pre; PTSD, post-traumatic stress disorder; VR, Virtual reality

**Supplementary Table 4.** Studies that considered firefighters’ job characteristics

| Year_Author | Where † | When † | Tailoring | How | |
| --- | --- | --- | --- | --- | --- |
|  |  |  |  | F/NF | I/G |
| **True-experimental study** | | | | | |
| 2019_Baek(10) | X | X | X | F | I |
| 2024_Kim(12) | O | * | X | F, NF | I |
| **Quasi-experimental study** | | | | | |
| 2013_Nam(13) | O | O | X | F | G |
| 2014_Park(15) | O | O | X | F | G |
| 2015_Shim(16) | NR | NR | O | F | G |
| 2016_Baek(17) | O | X | X | F | G |
| 2016_Lee(18) | X | X | X | F | I |
| 2017_Paik(8) | O | O | X | F | G |
| 2018_Han(9) | ** | ** | O | F | I, G |
| 2020A_Lee(19) | O | O | X | F | G |
| 2022_Hur(20) | NR | NR | X | NF | NR |
| 2023A_Kim(22) | O | O | X | F, NF | G |
| **Pre-experimental study** | | | | | |
| 2014_Chung(23) | X | X | O | F | I |
| 2016_Kim(24) | O | O | X | F | G |
| 2017_Kim(25) | O | O | O | F | I, G |
| 2017_Mo(26) | O | O | O | F | I, G |
| 2017_Lee(27) | NR | NR | X | F | G |
| 2018_Lee(28) | X | X | X | F | G |
| 2018_Shin(29) | X | X | O | F | I, G |
| 2019_Park(30) | X | X | X | F | G |
| 2020B_Lee(31) | O | O | O | F | I |
| 2020_Kim(32) | NR | NR | X | F | G |
| 2020_Jang(33) | NR | NR | O | F, NF | I, G |
| 2020_Won(34) | O | O | O | F | I, G |
| 2021_Jang(35) | X | NR | X | F | G |
| 2022_Park(36) | X | X | X | F | G |
| 2022_Cha(37) | X | X | X | F | G |
| 2022_Lee(39) | O | O | X | F | G |
| 2023B_Kim(41) | X | X | O | F | G |
| **Mixed-method design study** | | | | | |
| 2018_Yook(42) | NR | NR | O | F | I, G |
| 2019B_Kim(44) | X | X | O | F | G |
| 2019A_Kim(45) | X | X | X | F | I |
| 2023_Bak(46) | O | O | X | F | G |
| 2023_Kwak(47) | X | X | X | NF | G |
| **Qualitative study** | | | | | |
| 2022_Yoon(48) | O | O | X | F | G |

* Participants participate on their own time

** Depending on the program configuration, O or X

Where (Was the intervention conducted in the fire station?); When (Was the intervention conducted during work hours?)

F, Face-to-face; G, Group; I, Individual; NF, Non-face-to-face; NR, Not reported

Reference

1. Rosenbloom JL, Ginther DK, Juhl T, Heppert JA. The Effects of Research & Development Funding on Scientific Productivity: Academic Chemistry, 1990-2009. PLoS One. 2015;10(9):e0138176.

2. Kim I, Kim S. Police, Firefighter, and Coast Guard Mental Health Project Integration and Operation Plan Study (2017) [Internet]. KOREAN NATIONAL POLICE AGENCY; [cited 2024 Feb 29]. Available from: https://www.riss.kr/link?id=E1659915&ssoSkipYN=Y

3. Kang UI, Jung EK. Suicidal ideation among firefighters: A systematic literature review. The Korean Journal of Emergency Medical Services. 2023 Apr;27(1):7–18.

4. Lee JH, Lee D, Kim J, Jeon K, Sim M. Duty-Related Trauma Exposure and Posttraumatic Stress Symptoms in Professional Firefighters. J Trauma Stress. 2017 Apr;30(2):133–41.

5. Kim JM, Suh BS, Jung KY, Kim DI, Kim WS, Cho HS, et al. The study for musculoskeletal symptoms and job stress in firemen. Journal of Korean Society of Occupational and Environmental Hygiene. 2007;17(2):111–9.

6. Hulett DM, Bendick M, Thomas SY, Moccio F. A National Report Card on Women in Firefighting. International Association of Women in Fire & Emergency Services; 2008. 13 p.

7. Park J, Ahn YS, Kim MG. Pregnancy, childbirth, and puerperium outcomes in female firefighters in Korea. Ann Occup Environ Med. 2020 Jan 31;32:e8.

8. Paik Y woo, Choi M sun. The Effects of Group Arts Psychotherapy on Job Stress and Stress Coping Style for Fire-Fighting Officers. Korean Journal of Arts Therapy. 2017;17(1):157–73.

9. Han D, Bae J. Effects of a Post-traumatic Stress Disorder Management Program on Firefighters’ Post-traumatic Stress and Depression. Journal of Korean Academy of Psychiatric and Mental Health Nursing. 2018;27(3):240–51.

10. Baek JH, Heo JY, Fava M, Mischoulon D, Choi KW, Na EJ, et al. Effect of Korean Red Ginseng in individuals exposed to high stress levels: a 6-week, double-blind, randomized, placebo-controlled trial. J Ginseng Res. 2019 Jul;43(3):402–7.

11. Geerlings MI, Jonker C, Bouter LM, Adèr HJ, Schmand B. Association Between Memory Complaints and Incident Alzheimer’s Disease in Elderly People With Normal Baseline Cognition. AJP. 1999 Apr;156(4):531–7.

12. Kim JB, Xiang YY, Kim CB, Seo D, Song W, Lee H, et al. Improving Cardiorespiratory and Muscular Function of Korean Firefighters: A Kinect-Based Mixed Reality Device Exercise Intervention Randomized Control Trial. J Occup Environ Med. 2024 Feb 1;66(2):118–22.

13. Nam CY, Kim HS, Kwon SH. Effects of a Stress Management Program Providing Cognitive Behavior Therapy on Problem-focused Coping, Job Stress, and Depression in Firefighters. Journal of Korean Academy of Psychiatric and Mental Health Nursing. 2013;22(1):12–21.

14. Parker DF, DeCotiis TA. Organizational determinants of job stress. Organizational Behavior and Human Performance. 1983 Oct 1;32(2):160–77.

15. Chanseok P. Redesigning education programs for alleviating disaster response officials’ stressㆍPTSD and it’s empirical analysis for effectiveness. Journal of Korea Safety Management & Science. 2014;16(4):147–57.

16. Shim GS, Kim NS, Bang SH. Effects of Short-Term Music Therapy in Fire Fighters with Post-Traumatic Stress Risk. Journal of Korea Academia-Industrial cooperation Society. 2015;16(6):4040–7.

17. Baek ML, Kim YR. Effect of Music Listening on Convergent Job Stress of Fire-Fighter. Journal of the Korea Convergence Society. 2016;7(6):99–104.

18. Lee Y, Suk KJ, Bang CH, Lee JK, Huh YS, Park E. Effects of Wearing Firefighter Protective Clothing with Heavy Personal Protective Equipment on Physical, Biochemical, and Perceived Fatigue Parameters in Firefighting Officials. Journal of Wellness. 2016;11(1):195–206.

19. Lee GC, Park KJ. The Effect of Integrative Art Therapy Using Semantic Therapy on the Meaning of Life of New Fire Officials. Journal of The Korean Society of Industry Convergence. 2020;23(1):1–8.

20. Hur M, Jung I. Development of the Educational Program for Fire Officials’ Peer Psychological Support. Fire Science and Engineering. 2022;36(3):71–81.

21. Hwang J. self-reflection scale development and a path model of self-reflection to well-being [Internet]. [부천]: Catholic University Graduate School; 2011. Available from: https://m.riss.kr/link?id=T12457082

22. Kim EA, Cho KJ. Comparing the Effectiveness of Two New CPR Training Methods in Korea: Medical Virtual Reality Simulation and Flipped Learning. Iran J Public Health. 2023 Jul;52(7):1428–38.

23. Chung YH, Kim NH, Kim DH, Bae JH, Kwon JS, Jang JH, et al. The Psychological Characteristics and Functional Magnetic Resonance Imaging Findings in Firefighters with Partial Posttraumatic Stress Disorder and the Effect of Treatment by Using Eye Movement Desensitization and Reprocessing. JOURNAL OF THE KOREAN NEUROPSYCHIATRIC ASSOCIATION. 2014;53(2):122–33.

24. Kim EK, Park CS. A Study on the CISD Effectiveness. Crisisonomy. 2016;12(4):185–93.

25. Kim JJ, Choi TY, Kim SY, Park JH. The Effect of Mental Health Improvement Programs for Firefighters. Anxiety and Mood. 2017;13(1):17–24.

26. Mo JM, Yee BJ, Kwak M, Kang SM, Kim MK, Bae CH. The Development and Effectiveness of an Integrated Management Program for PTSD among Firefighters. Crisisonomy. 2017;13(7):17–33.

27. Lee MN, Na OH. Pre-to-Post Recognition of Trauma Group Education for Fire fighters. Asia-pacific Journal of Multimedia Services Convergent with Art, Humanities, and Sociology. 2017;7(10):413–21.

28. Lee I, Min YC, Yoo YG. Effects of Mind Subtraction Meditation Program on the Posttraumatic Growth and Resilience of 119 Emergency Medical Services Personnel. Fire Science and Engineering. 2018;32(5):95–104.

29. Shin J, Sim M, Lee J, Lee DY, Jeon K, Oh S, et al. Stress Relief and Related Factors in Camp Program for Firefighters. stress. 2018;26(2):88–94.

30. Park CH, Kang J, An M, Park S. Effects of Forest Therapy Program on Stress levels and Mood State in Fire Fighters. Fire Science and Engineering. 2019;33(6):132–41.

31. Lee BJ, Cho DU, Lee S ho. A Study on the Reduction of Stress for Firefighters According to Shift Work Using Personalized Birds Sound. The Journal of Korean Institute of Communications and Information Sciences. 2020;45(4):715–21.

32. Kim C, Ji C, Kim S. Effects of a High-Intensity Interval Training Program on the Fitness and Performance of Firefighters. Fire Science and Engineering. 2020;34(2):97–102.

33. Jang EH, Hong Y, Kim Y, Lee S, Ahn Y, Jeong KS, et al. The Development of a Sleep Intervention for Firefighters: The FIT-IN (Firefighter’s Therapy for Insomnia and Nightmares) Study. Int J Environ Res Public Health. 2020 Nov 24;17(23):8738.

34. Won GH, Lee JH, Choi TY, Yoon S, Kim SY, Park JH. The effect of a mental health promotion program on Korean firefighters. Int J Soc Psychiatry. 2020 Nov;66(7):675–81.

35. Jang HS, Yoo E, Kim JH, Jin JS, Kim JS, Ryu DY. Analysis of Psychological and Physiological Changes According to the Experience of the Care Farm of Fire-fighters. Journal of Korean Society of Rural Planning. 2021;27(1):71–83.

36. Park S, Park BJ. Effects of Forest Healing Program in Urban Forest on the Mental Health of Fire Officials and Satisfaction. Journal of recreation and landscape. 2022;16(4):47–56.

37. Cha JG, Lee YM. The effect of forest-based trauma stress management program on job stress, PTSD, resilience, mental health, and salivary cortisol for firefighters. KAIS. 2022 Jan 31;23(1):401–13.

38. Kim S, Kim Y. Firefighters’ Life Satisfaction and Job Stress: Moderating Effects of Family Resilience and Social Support. Family and Family Therapy. 2017 Jan;25(4):815–37.

39. Lee CG, Kwon J, Park S, Ahn C, Seo DI, Song W, et al. Process and Outcome Evaluations of Interventions to Promote Voluntary Exercise Training Among South Korean Firefighters. Am J Mens Health. 2022;16(1):15579883221076897.

40. Lee CG, Middlestadt SE, Park S, Kwon J, Noh K, Seo D il, et al. Predicting Voluntary Exercise Training among Korean Firefighters: Using Elicitation Study and the Theory of Planned Behavior. International Journal of Environmental Research and Public Health. 2020 Jan;17(2):467.

41. Kim MH, Kim H, Yi I soo. Effects of a Meditation-Based Healing Program on Stress Level, Stress Vulnerability, and Sleep Quality of Fire Officers. Fire Science and Engineering. 2023;37(5):81–8.

42. Yook Y. The Effects of Mindfulness based Body-Psychological Exercise Program on the Reduction of Post-Traumatic Stress(PTSD) Symptoms in Fire Officials. Korean Society of Sport Psychology. 2018;29(4):57–73.

43. Han D, Jeon G, Tak J, Lee C, Lee G. A study of life maladjustment in college students: a focus on anxiety, depression, and somatoform disorder. Proceedings of the Annual Meeting of the Korean Psychological Association. 1992;447–62.

44. Kim J, Yi I soo, Yoo YG. Effects of a Mind Subtraction Meditation–Based Healing Program on the Ruminations and Posttraumatic Stress Disorder Symptoms of Firefighters. Fire Science and Engineering. 2019;33(5):118–29.

45. Kim DH, Jung JY, Kim DH, Lee JY. Effects of Wearing Nomex Body Cooling Garment inside Firefighting Protective Equipment on the Efficiency of Performance During Simulated Firefighters’ Tasks. The Korean Society of Living Environmental System. 2019;26(1):9–24.

46. Bak J. The Effects of Simsang-Poetry Therapy on the Mental Health of Firefighters. The Journal of Humanities and Social science. 2023;14(3):101–16.

47. Kwak M, Im M. Developing and Evaluating an Online Post-Traumatic Growth Program for Firefighters. Issues Ment Health Nurs. 2023 Jul;44(7):663–72.

48. Yoon HS, Yoon H. A Case Study on the Application of the Happy Arts Therapy Program to Prevent Mental Health Problems of Fire-fighting Officers. Official Journal of the Koeran Society of Dance Science. 2022;39(1):1–27.
